# 4-[(5-Chloro-2-hy­droxy­benzyl­idene)amino]-3-ethyl-1*H*-1,2,4-triazole-5(4*H*)-thione

**DOI:** 10.1107/S1600536814008320

**Published:** 2014-04-18

**Authors:** Cai-Xia Yuan, Xu-Mei Yao, Miao-Li Zhu, Hong-Mei Zhu

**Affiliations:** a Institute of Molecular Science, Key Laboratory of Chemical Biology and Molecular Engineering of the Education Ministry, Shanxi University, Taiyuan, Shanxi 030006, People’s Republic of China; b College of Chemistry & Chemical Engineering, Shanxi University, Taiyuan, Shanxi 030006, People’s Republic of China

## Abstract

The title compound, C_11_H_11_ClN_4_OS, crystallizes with two mol­ecules, *A* and *B*, in the asymmetric unit in which the dihedral angles between the triazole and benzene rings are 54.6 (3) and 56.0 (3)°. Both mol­ecules feature an intra­molecular O—H⋯N hydrogen bond, which generates an *S*(6) ring. In the crystal, *A*–*B* dimers are linked by pairs of weak C—H⋯S hydrogen bonds along with π–π stacking inter­actions between the triazole rings [centroid–centroid separations = 3.631 (3) and 3.981 (4)Å]. N—H⋯S hydrogen bonds link the dimers into [100] chains, which feature *R*_2_^2^(8) loops.

## Related literature   

For background to 1,2,4-triazoles fused to Schiff bases, see: Sumangala *et al.* (2013 ▶); Brandt *et al.* (2007 ▶). For related structures, see: Pannu *et al.* (2011 ▶); Wu *et al.* (2012 ▶).
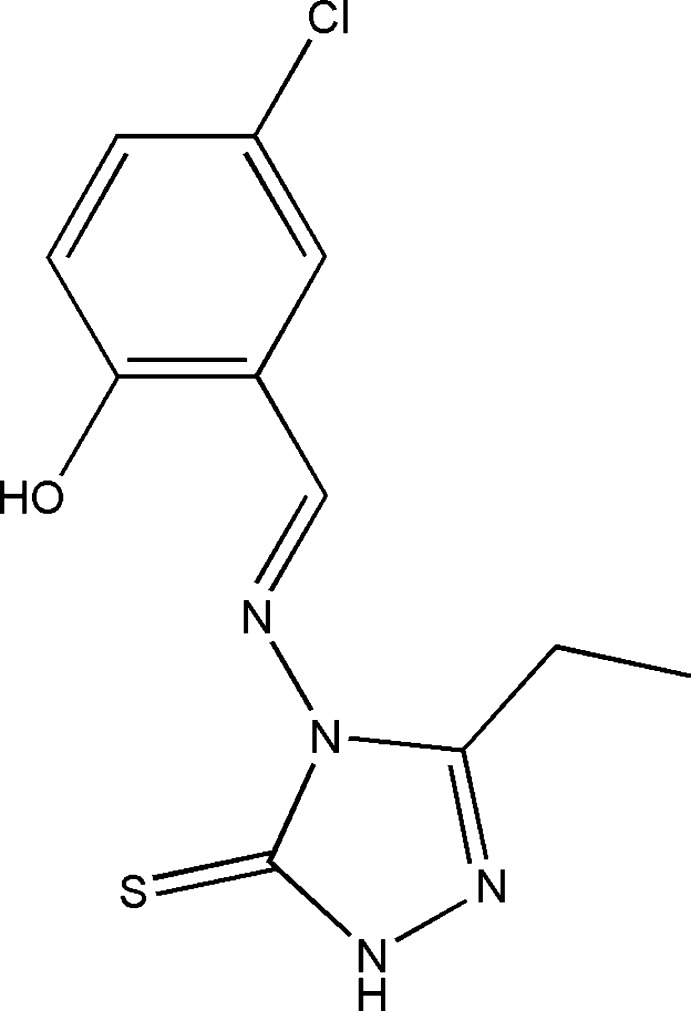


## Experimental   

### 

#### Crystal data  

C_11_H_11_ClN_4_OS
*M* *_r_* = 282.75
Monoclinic, 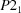

*a* = 6.297 (3) Å
*b* = 16.418 (8) Å
*c* = 12.290 (6) Å
β = 90.997 (7)°
*V* = 1270.2 (10) Å^3^
*Z* = 4
Mo *K*α radiation
μ = 0.46 mm^−1^
*T* = 298 K
0.20 × 0.20 × 0.15 mm

#### Data collection  

Bruker SMART APEX CCD diffractometer
Absorption correction: multi-scan (*SADABS*; Bruker, 2000 ▶) *T*_min_ = 0.914, *T*_max_ = 0.935
14098 measured reflections
2451 independent reflections
1924 reflections with *I* > 2σ(*I*)
*R*_int_ = 0.060

#### Refinement  

*R*[*F* ^2^ > 2σ(*F* ^2^)] = 0.037
*wR*(*F* ^2^) = 0.084
*S* = 1.03
2451 reflections
329 parameters
1 restraint
H-atom parameters constrained
Δρ_max_ = 0.17 e Å^−3^
Δρ_min_ = −0.27 e Å^−3^
Absolute structure: Flack (1983 ▶), 2274 Friedel pairs
Absolute structure parameter: 0.03 (10)

### 

Data collection: *SMART* (Bruker, 2000 ▶); cell refinement: *SAINT* (Bruker, 2000 ▶); data reduction: *SAINT*; program(s) used to solve structure: *SHELXS97* (Sheldrick, 2008 ▶); program(s) used to refine structure: *SHELXL97* (Sheldrick, 2008 ▶); molecular graphics: *SHELXTL* (Sheldrick, 2008 ▶); software used to prepare material for publication: *SHELXTL*.

## Supplementary Material

Crystal structure: contains datablock(s) I. DOI: 10.1107/S1600536814008320/hb7205sup1.cif

Structure factors: contains datablock(s) I. DOI: 10.1107/S1600536814008320/hb7205Isup2.hkl

Supporting information file. DOI: 10.1107/S1600536814008320/hb7205Isup3.cml

CCDC reference: 997034

Additional supporting information:  crystallographic information; 3D view; checkCIF report

## Figures and Tables

**Table 1:** Hydrogen-bond geometry (Å, °)

*D*—H⋯*A*	*D*—H	H⋯*A*	*D*⋯*A*	*D*—H⋯*A*
N1—H1⋯S2^i^	0.86	2.43	3.287 (4)	177
N5—H5*A*⋯S1^ii^	0.86	2.44	3.300 (4)	176
O1—H1*A*⋯N4	0.82	1.99	2.693 (5)	143
O2—H2⋯N8	0.82	1.99	2.699 (5)	144
C15—H15*A*⋯S1	0.96	3.01	3.922 (6)	160
C4—H4*B*⋯S2	0.96	2.87	3.805 (6)	164

Symmetry codes: (i) 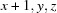; (ii) 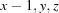.

**Fig. 1.**
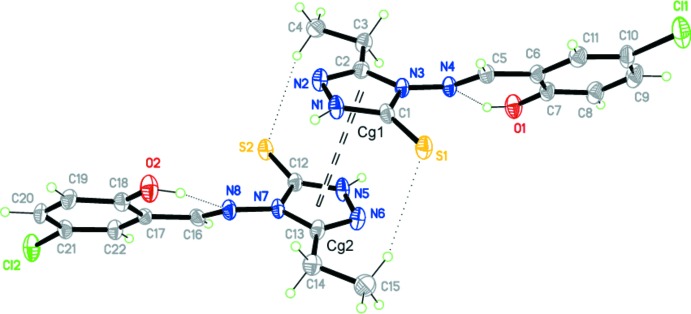
The view of the structure of (I) with displacement ellipsoids drawn at the 30% probability level. Dotted lines indicate hydrogen bonds and π–π interactions.

**Fig. 2.**
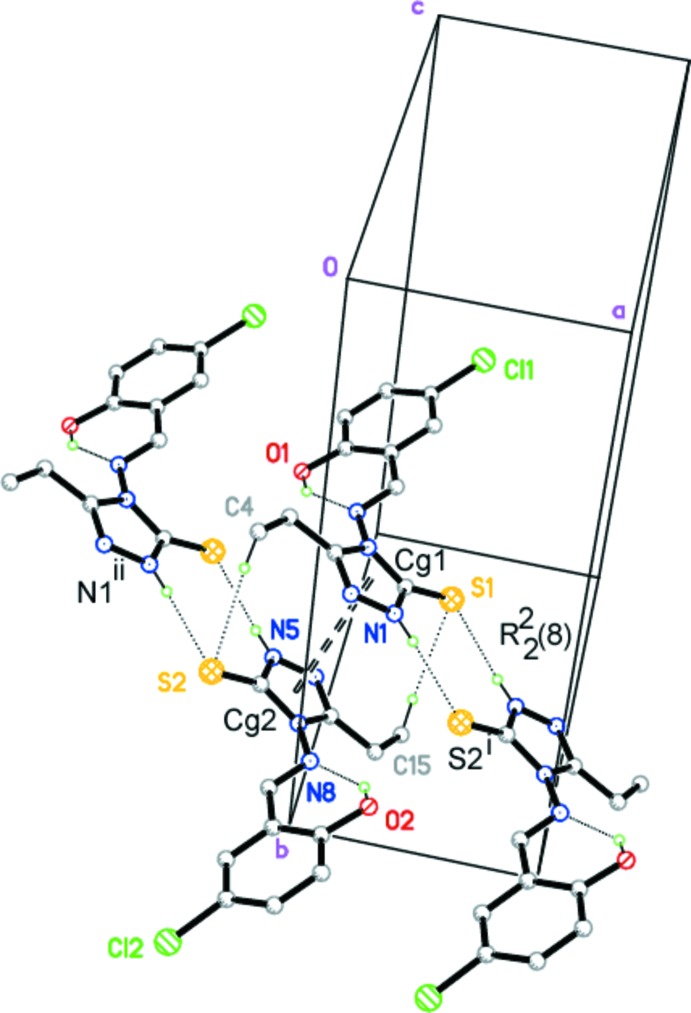
A part of the crystal structure I showing formation of a chain of R22(8) hydrogen-bonded rings and π–π stacking between Cg1 and Cg2 rings; Cg1: C1/C2/N1/N2/N3, Cg2: C12/C13/N5/N6/N7, Symmetry codes: i) x, y, z+1; ii) x, y, z-1.

## supplementary crystallographic information

### 1. Comment

The incorporation of the 1,2,4-triazole unit into Schiff base is of considerable current interest as complexes of 1,2,4-triazoles which are being developed for potential use in pharmaceutical and material applications (Sumangala *et al.* 2013; Brandt *et al.* 2007). Therefore, the title compound (I), has been synthesized and its crystal structure has been determinated.

The crystal structure is illustrated in Fig. 1 and the main geometric parameters of the commpound are listed in Table 1. The title compound (I) crystallizes in the monoclinic space group *P*2_1_ with two symmetry-independent molecules in the unit cell. The bond lengths of N4—C5 (1.275 Å) and N8—C16 (1.272 Å) confirm them as double bonds, which is similar to those reported in other Schiff bases (Pannu *et al.* 2011; Wu *et al.* 2012;). It is noticeable that the C—S bond length (1.670 Å) in the compound is close to C═S double bond, indicating that the compound exists in the thione form.

The packing arrangement in the crystal structure of (I) is shown in Fig. 2. As is a common feature of *o*-hydroxysalicylidene systems, the compound displays the strong intermolecular and intramolecular hydrogen bond between atoms N, O and S. The molecules of the compound is linked by an inversion-related pair of almost linear intermolecular hydrogen bonds N—H···S to form the cyclic centrosymmetric dimers characterized by an *R*_2_^2^(8) motif. The dimer is further held together by the π–π interactions between two rings (*Cg*1 and *Cg*2) in the crystal.

### 2. Experimental

0.5 mmol of 4-Amino-3-ethyl-1,2,4-triazole-5-thione was dissolved in 20 ml of ethanol with a constant stirring at 353 K. Then, 0.5 mmol of 5-chlorosalicylaldehyde in 10 ml of ethanol was added dropwise and the mixture solution was further refluxed for 2 h. The resulting yellow solution was filtered and the filtrate was left to stand at room temperature. The yellow blocks of the compound (I) were received from the filtrate with slowly evaporating solvent for a few days. Yield: 70%. Anal. Calcd. for C_11_H_11_ClN_4_OS: C 46.73, H 3.92, N 19.82%. Found: C 46.67, H 4.02, N 19.72%. IR (ν/cm^-1^): 3113, 3055, 2932, 1606, 1587, 1509, 1477, 1417, 1285, 1161, 1025, 965, 822, 657. UV/vis in DMSO, λ_max_/nm (ε 10^3^/*M*^-1^ cm^-1^): 260(19.94), 342(8.14).

### 3. Refinement

The H atoms bonded to C atoms were placed in calculated positions (C—H=0.96, 0.97 and 0.93 Å for C*sp*^3^, C*sp*^2^ and C*sp* atoms, respectively), assigned fixed *U*_iso_ values [*U*_iso_ H)=1.2 *U*_eq_ (C*sp*^2^) and 1.5*U*_eq_ (C*sp*^3^)] and treated as riding atoms. The H atoms attached to O and N atoms were found in the difference electron-density map and were refined isotropically, with O—H (0.82 Å) and N—H (0.86 Å) bond lengths.

### Crystal data

**Table:**

C_11_H_11_ClN_4_OS	*F*(000) = 584
*M**_r_* = 282.75	*D*_x_ = 1.478 Mg m^−^^3^
Monoclinic, *P*2_1_	Mo *K*α radiation, λ = 0.71073 Å
Hall symbol: P 2yb	Cell parameters from 1893 reflections
*a* = 6.297 (3) Å	θ = 2.5–20.7°
*b* = 16.418 (8) Å	µ = 0.46 mm^−^^1^
*c* = 12.290 (6) Å	*T* = 298 K
β = 90.997 (7)°	Block, colorless
*V* = 1270.2 (10) Å^3^	0.20 × 0.20 × 0.15 mm
*Z* = 4	

### Data collection

**Table:**

Bruker SMART APEX CCD diffractometer	2451 independent reflections
Radiation source: fine-focus sealed tube	1924 reflections with *I* > 2σ(*I*)
Graphite monochromator	*R*_int_ = 0.060
ω scans	θ_max_ = 25.5°, θ_min_ = 1.7°
Absorption correction: multi-scan (*SADABS*; Bruker, 2000)	*h* = −7→7
*T*_min_ = 0.914, *T*_max_ = 0.935	*k* = −19→19
14098 measured reflections	*l* = −14→14

### Refinement

**Table:**

Refinement on *F*^2^	Secondary atom site location: difference Fourier map
Least-squares matrix: full	Hydrogen site location: inferred from neighbouring sites
*R*[*F*^2^ > 2σ(*F*^2^)] = 0.037	H-atom parameters constrained
*wR*(*F*^2^) = 0.084	*w* = 1/[σ^2^(*F*_o_^2^) + (0.0375*P*)^2^ + 0.183*P*] where *P* = (*F*_o_^2^ + 2*F*_c_^2^)/3
*S* = 1.03	(Δ/σ)_max_ < 0.001
2451 reflections	Δρ_max_ = 0.17 e Å^−^^3^
329 parameters	Δρ_min_ = −0.27 e Å^−^^3^
1 restraint	Absolute structure: Flack (1983), 2274 Friedel pairs
Primary atom site location: structure-invariant direct methods	Absolute structure parameter: 0.03 (10)

### Special details

**Table:**

Geometry. All e.s.d.'s (except the e.s.d. in the dihedral angle between two l.s. planes) are estimated using the full covariance matrix. The cell e.s.d.'s are taken into account individually in the estimation of e.s.d.'s in distances, angles and torsion angles; correlations between e.s.d.'s in cell parameters are only used when they are defined by crystal symmetry. An approximate (isotropic) treatment of cell e.s.d.'s is used for estimating e.s.d.'s involving l.s. planes.
Refinement. Refinement of *F*^2^ against ALL reflections. The weighted *R*-factor *wR* and goodness of fit *S* are based on *F*^2^, conventional *R*-factors *R* are based on *F*, with *F* set to zero for negative *F*^2^. The threshold expression of *F*^2^ > σ(*F*^2^) is used only for calculating *R*-factors(gt) *etc*. and is not relevant to the choice of reflections for refinement. *R*-factors based on *F*^2^ are statistically about twice as large as those based on *F*, and *R*- factors based on ALL data will be even larger.

### Fractional atomic coordinates and isotropic or equivalent isotropic displacement parameters (Å^2^)

**Table:**

	*x*	*y*	*z*	*U*_iso_*/*U*_eq_	
Cl1	0.3625 (2)	0.60813 (11)	1.00331 (11)	0.0760 (5)	
N1	0.2557 (6)	0.7267 (3)	0.2890 (3)	0.0444 (10)	
H1	0.3494	0.7463	0.2461	0.053*	
N2	0.0832 (7)	0.6828 (3)	0.2520 (3)	0.0438 (11)	
N3	0.0891 (6)	0.6948 (3)	0.4301 (3)	0.0347 (10)	
N4	0.0053 (6)	0.6921 (3)	0.5349 (3)	0.0377 (10)	
O1	−0.2784 (6)	0.7242 (3)	0.6907 (3)	0.0543 (11)	
H1A	−0.2346	0.7258	0.6283	0.081*	
S1	0.44562 (18)	0.78763 (8)	0.47087 (9)	0.0436 (3)	
C1	0.2662 (7)	0.7364 (3)	0.3962 (3)	0.0364 (11)	
C2	−0.0177 (8)	0.6646 (3)	0.3409 (4)	0.0377 (12)	
C3	−0.2158 (8)	0.6175 (3)	0.3463 (4)	0.0453 (12)	
H3A	−0.3226	0.6503	0.3816	0.054*	
H3B	−0.1909	0.5695	0.3908	0.054*	
C4	−0.3003 (8)	0.5911 (3)	0.2359 (4)	0.0518 (14)	
H4A	−0.1964	0.5578	0.2009	0.078*	
H4B	−0.3294	0.6383	0.1921	0.078*	
H4C	−0.4287	0.5605	0.2447	0.078*	
C5	0.1366 (8)	0.6718 (3)	0.6103 (4)	0.0393 (13)	
H5	0.2741	0.6570	0.5923	0.047*	
C6	0.0756 (8)	0.6715 (3)	0.7232 (4)	0.0368 (12)	
C7	−0.1230 (8)	0.6988 (3)	0.7578 (4)	0.0411 (13)	
C8	−0.1660 (8)	0.7003 (3)	0.8684 (4)	0.0523 (14)	
H8	−0.2956	0.7203	0.8918	0.063*	
C9	−0.0194 (9)	0.6725 (4)	0.9431 (4)	0.0572 (15)	
H9	−0.0502	0.6730	1.0168	0.069*	
C10	0.1759 (9)	0.6436 (3)	0.9084 (4)	0.0497 (14)	
C11	0.2243 (8)	0.6449 (3)	0.8005 (4)	0.0456 (14)	
H11	0.3576	0.6278	0.7785	0.055*	
Cl2	−0.3045 (2)	0.97311 (11)	−0.41889 (11)	0.0672 (5)	
N5	−0.2140 (6)	0.8673 (3)	0.2996 (3)	0.0477 (11)	
H5A	−0.3078	0.8485	0.3430	0.057*	
N6	−0.0421 (7)	0.9120 (3)	0.3347 (3)	0.0471 (11)	
N7	−0.0462 (6)	0.8961 (2)	0.1579 (3)	0.0360 (10)	
N8	0.0415 (6)	0.8959 (3)	0.0537 (3)	0.0404 (10)	
O2	0.3237 (5)	0.8558 (3)	−0.1000 (3)	0.0567 (10)	
H2	0.2853	0.8632	−0.0374	0.085*	
S2	−0.40057 (19)	0.80215 (8)	0.11776 (9)	0.0453 (3)	
C12	−0.2233 (7)	0.8553 (3)	0.1915 (4)	0.0365 (11)	
C13	0.0582 (8)	0.9277 (3)	0.2478 (4)	0.0389 (12)	
C14	0.2574 (8)	0.9764 (4)	0.2401 (4)	0.0488 (13)	
H14A	0.2321	1.0232	0.1935	0.059*	
H14B	0.3670	0.9434	0.2075	0.059*	
C15	0.3339 (9)	1.0054 (3)	0.3517 (4)	0.0591 (15)	
H15A	0.3540	0.9593	0.3988	0.089*	
H15B	0.2298	1.0412	0.3820	0.089*	
H15C	0.4659	1.0340	0.3448	0.089*	
C16	−0.0890 (8)	0.9128 (3)	−0.0232 (4)	0.0376 (13)	
H16	−0.2273	0.9275	−0.0064	0.045*	
C17	−0.0282 (8)	0.9097 (3)	−0.1362 (4)	0.0363 (12)	
C18	0.1690 (8)	0.8807 (3)	−0.1703 (4)	0.0400 (12)	
C19	0.2111 (8)	0.8760 (3)	−0.2799 (4)	0.0463 (14)	
H19	0.3395	0.8542	−0.3024	0.056*	
C20	0.0655 (8)	0.9030 (3)	−0.3562 (4)	0.0440 (14)	
H20	0.0955	0.8999	−0.4299	0.053*	
C21	−0.1263 (9)	0.9350 (3)	−0.3228 (4)	0.0423 (13)	
C22	−0.1739 (8)	0.9368 (3)	−0.2148 (4)	0.0417 (13)	
H22	−0.3053	0.9563	−0.1935	0.050*	

### Atomic displacement parameters (Å^2^)

**Table:**

	*U*^11^	*U*^22^	*U*^33^	*U*^12^	*U*^13^	*U*^23^
Cl1	0.0791 (11)	0.1054 (14)	0.0429 (8)	−0.0219 (10)	−0.0152 (7)	0.0181 (8)
N1	0.041 (2)	0.065 (3)	0.027 (2)	−0.005 (2)	0.0082 (18)	0.003 (2)
N2	0.042 (3)	0.061 (3)	0.029 (2)	−0.002 (2)	0.0026 (19)	−0.002 (2)
N3	0.036 (2)	0.044 (3)	0.024 (2)	0.000 (2)	0.0078 (17)	0.0003 (18)
N4	0.040 (2)	0.050 (3)	0.024 (2)	0.001 (2)	0.0079 (18)	0.0020 (19)
O1	0.048 (2)	0.078 (3)	0.037 (2)	0.010 (2)	0.0084 (18)	0.002 (2)
S1	0.0429 (7)	0.0589 (8)	0.0293 (6)	−0.0055 (6)	0.0057 (5)	−0.0008 (6)
C1	0.038 (3)	0.045 (3)	0.026 (3)	0.008 (2)	0.007 (2)	0.006 (2)
C2	0.039 (3)	0.041 (3)	0.033 (3)	0.009 (2)	0.000 (2)	0.000 (2)
C3	0.046 (3)	0.052 (3)	0.039 (3)	0.000 (3)	0.005 (2)	−0.004 (2)
C4	0.060 (3)	0.046 (3)	0.049 (3)	−0.007 (3)	−0.008 (3)	0.000 (3)
C5	0.043 (3)	0.043 (3)	0.032 (3)	−0.002 (2)	0.009 (2)	0.002 (2)
C6	0.042 (3)	0.041 (3)	0.027 (3)	−0.006 (2)	0.007 (2)	0.001 (2)
C7	0.049 (3)	0.046 (3)	0.029 (3)	−0.010 (3)	0.010 (2)	−0.002 (2)
C8	0.055 (3)	0.066 (4)	0.037 (3)	−0.005 (3)	0.014 (3)	−0.004 (3)
C9	0.069 (4)	0.072 (4)	0.031 (3)	−0.024 (3)	0.017 (3)	−0.005 (3)
C10	0.062 (4)	0.060 (4)	0.026 (3)	−0.021 (3)	−0.003 (2)	0.004 (2)
C11	0.048 (3)	0.050 (3)	0.039 (3)	−0.007 (3)	0.007 (2)	−0.001 (3)
Cl2	0.0642 (9)	0.1033 (13)	0.0339 (7)	0.0065 (9)	−0.0054 (6)	0.0041 (8)
N5	0.045 (3)	0.073 (3)	0.025 (2)	−0.002 (2)	0.0093 (19)	0.002 (2)
N6	0.045 (3)	0.066 (3)	0.030 (2)	0.000 (2)	0.003 (2)	−0.006 (2)
N7	0.040 (2)	0.048 (3)	0.020 (2)	0.006 (2)	0.0032 (17)	0.0036 (18)
N8	0.044 (2)	0.050 (3)	0.027 (2)	0.002 (2)	0.0106 (19)	0.0017 (19)
O2	0.051 (2)	0.082 (3)	0.037 (2)	0.018 (2)	0.0077 (17)	0.005 (2)
S2	0.0447 (7)	0.0605 (9)	0.0308 (6)	−0.0031 (7)	0.0049 (5)	0.0026 (6)
C12	0.036 (3)	0.045 (3)	0.029 (3)	0.005 (2)	0.005 (2)	0.002 (2)
C13	0.039 (3)	0.050 (3)	0.028 (3)	0.008 (2)	0.004 (2)	−0.004 (2)
C14	0.052 (3)	0.053 (3)	0.042 (3)	0.001 (3)	0.006 (2)	−0.002 (3)
C15	0.064 (4)	0.058 (4)	0.054 (3)	−0.014 (3)	−0.011 (3)	0.001 (3)
C16	0.041 (3)	0.037 (3)	0.034 (3)	0.001 (2)	0.009 (2)	0.006 (2)
C17	0.044 (3)	0.034 (3)	0.031 (3)	−0.009 (2)	0.010 (2)	−0.002 (2)
C18	0.045 (3)	0.042 (3)	0.034 (3)	0.001 (2)	0.007 (2)	0.001 (2)
C19	0.052 (3)	0.048 (3)	0.039 (3)	−0.004 (3)	0.017 (3)	−0.008 (3)
C20	0.056 (3)	0.051 (3)	0.025 (3)	−0.003 (3)	0.011 (2)	−0.005 (2)
C21	0.051 (3)	0.047 (3)	0.029 (3)	−0.005 (3)	−0.003 (2)	0.002 (2)
C22	0.040 (3)	0.052 (3)	0.034 (3)	0.003 (2)	0.005 (2)	0.001 (2)

### Geometric parameters (Å, º)

**Table:**

Cl1—C10	1.741 (5)	Cl2—C21	1.732 (5)
N1—C1	1.327 (6)	N5—C12	1.342 (6)
N1—N2	1.375 (6)	N5—N6	1.371 (5)
N1—H1	0.8600	N5—H5A	0.8600
N2—C2	1.308 (7)	N6—C13	1.277 (7)
N3—C2	1.370 (6)	N7—C12	1.370 (6)
N3—C1	1.378 (6)	N7—C13	1.377 (6)
N3—N4	1.401 (5)	N7—N8	1.403 (5)
N4—C5	1.275 (6)	N8—C16	1.272 (6)
O1—C7	1.335 (6)	O2—C18	1.355 (5)
O1—H1A	0.8200	O2—H2	0.8200
S1—C1	1.670 (5)	S2—C12	1.672 (5)
C2—C3	1.470 (7)	C13—C14	1.492 (7)
C3—C4	1.512 (6)	C14—C15	1.522 (6)
C3—H3A	0.9700	C14—H14A	0.9700
C3—H3B	0.9700	C14—H14B	0.9700
C4—H4A	0.9600	C15—H15A	0.9600
C4—H4B	0.9600	C15—H15B	0.9600
C4—H4C	0.9600	C15—H15C	0.9600
C5—C6	1.446 (6)	C16—C17	1.448 (7)
C5—H5	0.9300	C16—H16	0.9300
C6—C11	1.392 (7)	C17—C22	1.394 (7)
C6—C7	1.402 (7)	C17—C18	1.400 (7)
C7—C8	1.390 (6)	C18—C19	1.380 (7)
C8—C9	1.369 (7)	C19—C20	1.373 (7)
C8—H8	0.9300	C19—H19	0.9300
C9—C10	1.392 (8)	C20—C21	1.386 (7)
C9—H9	0.9300	C20—H20	0.9300
C10—C11	1.366 (6)	C21—C22	1.366 (7)
C11—H11	0.9300	C22—H22	0.9300
			
C1—N1—N2	114.6 (4)	C12—N5—N6	114.2 (4)
C1—N1—H1	122.7	C12—N5—H5A	122.9
N2—N1—H1	122.7	N6—N5—H5A	122.9
C2—N2—N1	103.7 (4)	C13—N6—N5	104.2 (4)
C2—N3—C1	109.0 (4)	C12—N7—C13	108.8 (4)
C2—N3—N4	122.5 (4)	C12—N7—N8	127.5 (4)
C1—N3—N4	127.8 (4)	C13—N7—N8	122.8 (4)
C5—N4—N3	115.2 (4)	C16—N8—N7	114.8 (4)
C7—O1—H1A	109.5	C18—O2—H2	109.5
N1—C1—N3	102.4 (4)	N5—C12—N7	101.8 (4)
N1—C1—S1	128.8 (4)	N5—C12—S2	129.0 (4)
N3—C1—S1	128.8 (3)	N7—C12—S2	129.1 (3)
N2—C2—N3	110.3 (4)	N6—C13—N7	111.0 (5)
N2—C2—C3	125.7 (4)	N6—C13—C14	126.2 (5)
N3—C2—C3	124.0 (4)	N7—C13—C14	122.8 (4)
C2—C3—C4	113.3 (4)	C13—C14—C15	111.3 (4)
C2—C3—H3A	108.9	C13—C14—H14A	109.4
C4—C3—H3A	108.9	C15—C14—H14A	109.4
C2—C3—H3B	108.9	C13—C14—H14B	109.4
C4—C3—H3B	108.9	C15—C14—H14B	109.4
H3A—C3—H3B	107.7	H14A—C14—H14B	108.0
C3—C4—H4A	109.5	C14—C15—H15A	109.5
C3—C4—H4B	109.5	C14—C15—H15B	109.5
H4A—C4—H4B	109.5	H15A—C15—H15B	109.5
C3—C4—H4C	109.5	C14—C15—H15C	109.5
H4A—C4—H4C	109.5	H15A—C15—H15C	109.5
H4B—C4—H4C	109.5	H15B—C15—H15C	109.5
N4—C5—C6	121.2 (5)	N8—C16—C17	121.8 (5)
N4—C5—H5	119.4	N8—C16—H16	119.1
C6—C5—H5	119.4	C17—C16—H16	119.1
C11—C6—C7	119.0 (4)	C22—C17—C18	118.5 (5)
C11—C6—C5	118.0 (5)	C22—C17—C16	118.2 (5)
C7—C6—C5	122.9 (5)	C18—C17—C16	123.4 (5)
O1—C7—C8	116.4 (4)	O2—C18—C19	117.2 (4)
O1—C7—C6	124.1 (4)	O2—C18—C17	122.9 (4)
C8—C7—C6	119.5 (5)	C19—C18—C17	119.9 (5)
C9—C8—C7	120.6 (5)	C20—C19—C18	120.7 (5)
C9—C8—H8	119.7	C20—C19—H19	119.7
C7—C8—H8	119.7	C18—C19—H19	119.7
C8—C9—C10	119.8 (5)	C19—C20—C21	119.7 (5)
C8—C9—H9	120.1	C19—C20—H20	120.1
C10—C9—H9	120.1	C21—C20—H20	120.1
C11—C10—C9	120.4 (5)	C22—C21—C20	120.2 (5)
C11—C10—Cl1	119.8 (4)	C22—C21—Cl2	120.3 (4)
C9—C10—Cl1	119.9 (4)	C20—C21—Cl2	119.5 (4)
C10—C11—C6	120.6 (5)	C21—C22—C17	120.9 (5)
C10—C11—H11	119.7	C21—C22—H22	119.5
C6—C11—H11	119.7	C17—C22—H22	119.5
			
C1—N1—N2—C2	0.1 (6)	C12—N5—N6—C13	−0.4 (6)
C2—N3—N4—C5	137.3 (5)	C12—N7—N8—C16	52.0 (6)
C1—N3—N4—C5	−53.0 (7)	C13—N7—N8—C16	−139.5 (5)
N2—N1—C1—N3	0.8 (5)	N6—N5—C12—N7	−0.6 (5)
N2—N1—C1—S1	−178.1 (4)	N6—N5—C12—S2	177.7 (4)
C2—N3—C1—N1	−1.3 (5)	C13—N7—C12—N5	1.3 (5)
N4—N3—C1—N1	−172.1 (4)	N8—N7—C12—N5	171.1 (4)
C2—N3—C1—S1	177.6 (4)	C13—N7—C12—S2	−177.0 (4)
N4—N3—C1—S1	6.8 (7)	N8—N7—C12—S2	−7.2 (7)
N1—N2—C2—N3	−0.9 (5)	N5—N6—C13—N7	1.3 (5)
N1—N2—C2—C3	180.0 (5)	N5—N6—C13—C14	179.0 (5)
C1—N3—C2—N2	1.5 (6)	C12—N7—C13—N6	−1.7 (6)
N4—N3—C2—N2	172.8 (4)	N8—N7—C13—N6	−172.1 (4)
C1—N3—C2—C3	−179.4 (5)	C12—N7—C13—C14	−179.5 (5)
N4—N3—C2—C3	−8.0 (7)	N8—N7—C13—C14	10.1 (7)
N2—C2—C3—C4	−0.8 (7)	N6—C13—C14—C15	0.9 (8)
N3—C2—C3—C4	−179.8 (5)	N7—C13—C14—C15	178.4 (4)
N3—N4—C5—C6	176.1 (4)	N7—N8—C16—C17	−175.6 (4)
N4—C5—C6—C11	177.0 (5)	N8—C16—C17—C22	−173.7 (5)
N4—C5—C6—C7	−4.9 (8)	N8—C16—C17—C18	6.4 (8)
C11—C6—C7—O1	−178.7 (5)	C22—C17—C18—O2	177.7 (5)
C5—C6—C7—O1	3.2 (8)	C16—C17—C18—O2	−2.5 (8)
C11—C6—C7—C8	1.2 (8)	C22—C17—C18—C19	−3.0 (8)
C5—C6—C7—C8	−176.9 (5)	C16—C17—C18—C19	176.8 (5)
O1—C7—C8—C9	177.5 (5)	O2—C18—C19—C20	−177.5 (5)
C6—C7—C8—C9	−2.4 (8)	C17—C18—C19—C20	3.2 (8)
C7—C8—C9—C10	0.9 (9)	C18—C19—C20—C21	−0.4 (8)
C8—C9—C10—C11	1.8 (8)	C19—C20—C21—C22	−2.5 (8)
C8—C9—C10—Cl1	−179.9 (4)	C19—C20—C21—Cl2	177.2 (4)
C9—C10—C11—C6	−3.0 (8)	C20—C21—C22—C17	2.6 (8)
Cl1—C10—C11—C6	178.7 (4)	Cl2—C21—C22—C17	−177.1 (4)
C7—C6—C11—C10	1.5 (8)	C18—C17—C22—C21	0.2 (8)
C5—C6—C11—C10	179.7 (5)	C16—C17—C22—C21	−179.7 (5)

### Hydrogen-bond geometry (Å, º)

**Table:**

*D*—H···*A*	*D*—H	H···*A*	*D*···*A*	*D*—H···*A*
N1—H1···S2^i^	0.86	2.43	3.287 (4)	177
N5—H5*A*···S1^ii^	0.86	2.44	3.300 (4)	176
O1—H1*A*···N4	0.82	1.99	2.693 (5)	143
O2—H2···N8	0.82	1.99	2.699 (5)	144
C15—H15*A*···S1	0.96	3.01	3.922 (6)	160
C4—H4*B*···S2	0.96	2.87	3.805 (6)	164
*Cg*1···*Cg*2			3.631 (3)	
*Cg*3···*Cg*4^iii^			3.981 (4)	

Symmetry codes: (i) *x*+1, *y*, *z*; (ii) *x*−1, *y*, *z*; (iii) *x*, *y*, *z*+1.
